# 1. An usual case of osteomyelitis in rheumatology

**DOI:** 10.1093/rap/rky031

**Published:** 2018-09-20

**Authors:** Naval Mendiratta, Dhiren Raval, Wasim Kazi, Gaytri Ekbote

**Affiliations:** 1 Rheumatology, Fortis Memorial Research Institute, Haryana, INDIA; 2 Rheumatology, Medanta the Medcity Hospital, Haryana, INDIA

**Introduction:** We hereby present a case of a 13 year old male, who was admitted in our hospital with complaints of multiple pustular lesions over the neck, scalp, face along with polyarthritis and new sites of osteomyelitis over the elbows, knees and wrist. The osteitis were so debilitating that the patient was bed bound and had already undergone seven debridements without much benefit. He was finally diagnosed with chronic recurrent multifocal osteomyelitis/pyogenic arthritis with pyoderma gangrenosum and acne (PAPA Syndrome) (genetic testing could not be performed). We decided to put forward this case as non-bacterial osteomyelitis is a very rare entity and if treated early, can help reduce the morbidity adolescents face with the diagnostic dilemma. Also, our patient was refractory to standard treatment as the lesions kept relapsing on tapering steroids,with DMARDS proving to be partially efficacious only. Hence he had to be commenced on TNF blockers (adalimumab) to which he showed a dramatic response and is now able to resume his normal life.

**Case description:** A 13-year-old male came to our hospital with history of multiple joint pains and swelling along with multiple skin lesions involving the scalp, neck and face for ten months. It started in February 2016 when patient was first admitted in Nepal with high grade fever along with pustular skin lesions over the face, scalp, neck. He was diagnosed as having a folliculitis abscess. He underwent incision and drainage and was treated with a course of antibiotics and anti-inflammatory treatment. He felt better and was discharged. He was able to resume his normal activities before he got another episode of pustular lesions, fever and left elbow swelling in May 2016. Culture did not show any growth and patient was treated with a course of antibiotics, anti fungals along with anti-inflammatory and discharged. Two months later in August 2016, patient was again admitted with involvement of new site which was the left knee. The deep-seated abscess was drained again and the repeat culture again did not show any growth. As the inflammatory markers were very high (ESR 97 CRP 126), he was repeated with different set of antibiotics. The quiescent period didn’t last too long and again in the month of September he got admitted with two new sites this time involving the right wrist and left elbow. The deep-seated abscess were extremely painful and he was unable to extend his arm. He underwent debridement for both sites and given a course of extended antibiotics for 14 days. Patient continued feeling unwell and in November 2016 he had another episode involving left knee effusion with restriction of movements. He had become bed bound due to severe pain and was unable to extend his knee. Before planning any further surgery, patient was air lifted to India and was admitted in our hospital to the paediatrics department. On examination, patient had a grossly swollen left knee along with multiple pustular lesions over the face, scalp and neck. There was a large non healing wound over the right wrist (3.2 cms) and right elbow which was deep seated causing restriction of activities of the joints. Laboratory evaluation showed high CRP 266 and leucocytosis 21000. MRI of the right wrist and elbow showed localised collection of radial and volar aspect of the wrist, associated tenosynovitis, focal osteomyelitis of scaphoid and trapezium, right elbow septic arthritis with focal collection at olecranon process, osteomyelitis in the adjoining olecranon process of the ulna and proximal radius. It was likely septic. Patient was referred to a paediatric orthopaedic surgeon and he underwent right elbow debridement (seventh overall). Since the left knee was still swollen, a rheumatology review was taken for possibility of juvenile idiopathic arthritis. Finally looking at all the picture with multiple focal sites of osteomyelitis and sterile culture, painful pustules and persistently raised inflammatory markers we decided against sepsis. A working diagnosis of chronic recurrent multifocal osteomyelitis was considered. Since there were no genetic markers available for confirmation, we had to go with our strong suspicion for the diagnosis. Since he continued to relapse on anti-inflammatory drugs, we started him on oral prednisolone (1mg/kg). Within two days, there was resolution of knee inflammation. The wrist and elbow lesions started healing and the fever subsided. The patient was discharged after three days and he followed us up in outpatient department. The joints showed a dramatic response but on tapering wysolone to less than 20 mg, he started having new painful pustules over the nose, cheeks and face. Tab methotrexate was started at 15 mg once a week as a steroid sparing drug, which was later increased to 20 mg. We managed to bring down the steroids to 10 mg but he again had a relapse of skin lesions. The osteomyelitis sites at this stage were relatively stable. Subsequently colchicine and sulphasalazine were also added over the next 4-6 months and methotrexate was increased to 25 mg once a week. But we failed to bring down the steroid dose less than 7.5 mg. Since he was an adolescent and we didn’t want to affect his growth spurt with continuous use of steroids, we decided to go for TNF blockers. After the review of literature for refractory cases, we decided to go for the biosimilar adalimumab available in our country. As the screening for tuberculosis and viral hepatitis was negative, he was given 40 mg subcutaneous once in two weeks for next three months. The skin disease responded very well and the pustular lesions subsided. We stopped sulphasalazine and colchicine since both did not show much efficacy. Methotrexate was continued at 25 mg once a week and we managed to cut down prednisolone to 2.5 mg on alternate days. MRI of the knees, wrist and elbow were repeated after nine months of therapy which showed complete resolution of the osteomyelitis. Patient has been under our follow up and he is currently doing well. His last dose of adalimumab was six months back and he continues on methotrexate 25 mg once a week and prednisolone 2.5 mg twice a week. Although he does get few lesions over the scalp occasionally, but none of them are as painful as the earlier ones and usually subsides with a short course of naprosyn (3-5 days). He is now able to resume his studies which had taken a setback due to his illness last year.

**Discussion:** Chronic recurrent multifocal osteomyelitis (CRMO) is one of the rare diseases which has been reported under genetic and rare disease (GARD) information centre. It is classified under autoinflammatory syndromes, which includes other disease like SAPHO syndrome, PAPA syndrome which have a significant overlap and can only be distinguished from the genetic analysis. Since we did not perform genetic analysis of our patient, the confirmed diagnosis could not be labelled. However, if we follow the Bristol Criteria for CRMO it fits in completely. As seen with the other studies, CRMO has a chronic course in 57% of the patients and our patient was no exclusion. He still continues to experience mild skin flares even after one and a half years of treatment. Bisphosphonates have been recommended for the treatment but since he responded well to Steroids and TNF blockers we did not find the need to commence them. As seen in the literature, complete radiographic resolution may not be seen with all the patients in chronic phase, but our patient showed complete healing of the radiographic sites on follow up MRI. It gave us a clue that if we attack the disease aggressively at early stage, things can be reversed. It is an important lesson learnt as all abscesses/osteomyelitis are not infective especially if a patient is having recurrent ones with sterile pus culture. Average delay in diagnosis has been reported as ten months. But if we all are aware of this disease, it can help reduce the morbidity of the patients. So always get your rheumatologist on board for any doubts in diagnosis.

**Key Learning Points**: Not all the abscesses/osteomyelitis are infective. Always suspect non-bacterial osteomyelitis in patients who present with multiple site involvement. Do keep a rheumatologist on board if you are facing a diagnostic dilemma. We should treat the patients with CRMO aggressively as long term outcomes do vary.

**Disclosure: N. Mendiratta:** None. **D. Raval:** None. **W. Kazi:** None. **G. Ekbote:** None.

